# Expression of *N*-acetylglucosaminyltransferase V in endometrial cancer correlates with poor prognosis

**DOI:** 10.1038/sj.bjc.6604044

**Published:** 2007-10-30

**Authors:** E Yamamoto, K Ino, E Miyoshi, K Shibata, N Takahashi, H Kajiyama, A Nawa, S Nomura, T Nagasaka, F Kikkawa

**Affiliations:** 1 Department of Obstetrics and Gynecology, Nagoya University Graduate School of Medicine, 65 Tsurumai-cho, Showa-ku, Nagoya 466-8550, Japan; 2 Department of Molecular Biochemistry and Clinical Investigation, Osaka University Graduate School of Medicine, Suita, Japan; 3 Department of Pathology and Clinical Laboratories, Nagoya University Graduate School of Medicine, Nagoya, Japan

**Keywords:** *N*-acetylglucosaminyltransferase V, endometrial cancer, prognostic factor, progression-free survival (PFS)

## Abstract

*N*-acetylglucosaminyltransferase V (GnT-V) is an enzyme that catalyses *β*1–6 branching of *N*-acetylglucosamine on asparagine-linked oligosaccharides of cell proteins. The present study aimed to investigate GnT-V expression and its prognostic significance in endometrial cancer. *N*-acetylglucosaminyltransferase V expression was studied by immunohistochemistry in 74 surgically resected endometrial cancers, and the staining intensity was evaluated. High GnT-V expression in tumour cells was found in 43 (58.1%) of the 74 cases, and was positively correlated with advanced patient age, histological grade, and lymph vascular space involvement. Patients with high GnT-V expression had significantly impaired overall survival and progression-free survival (PFS) (*P*=0.0041 and *P*=0.0023, respectively) compared to patients with low expression of GnT-V. On multivariate analysis, GnT-V expression was an independent prognostic factor for PFS (*P*=0.0364). *β*1–6 branching of asparagine-linked oligosaccharides was also detected in GnT-V-positive endometrial cancer cells by leukoagglutinating phytohaemagglutinin (L_4_-PHA) staining, and the molecular size of the major glycoproteins recognised by L_4_-PHA was approximately 60–200 kDa by lectin blot analysis. These results suggested that high GnT-V expression was correlated with an unfavourable clinical outcome, and that GnT-V is involved in the malignant potential of endometrial cancer by increasing the synthesis of *β*1–6 branching of asparagine-linked oligosaccharides.

The glycosylation of cell-surface glycoproteins is widely accepted to play a key role in a variety of specific biological interactions (Hakomori, 1989). In particular, branching of asparagine-linked oligosaccharides is shown to regulate metastatic potential in cancer cells (Pierce *et al*, 1997). Among the several patterns of branching, *β*1–6 branching of *N*-acetylglucosamine to *α*-D-6 mannnoside enhances metastasis in experimental cancer models of mice (Dennis *et al*, 1987). *N*-acetylglucosaminyltransferase V (GnT-V, EC 4.1.15) catalyses this branching and is most strongly linked to tumour invasion and metastasis. In human cancers, several studies have shown that high activity or expression of GnT-V was associated with poor prognosis in human colorectal cancer (Murata *et al*, 2000) and breast cancer (Fernandes *et al*, 1991). On the other hand, GnT-V is expressed in normal human lung and low expression of GnT-V in non-small cell lung cancer is associated with poor prognosis (Dosaka-Akita *et al*, 2004). Thus, GnT-V expression and its functional and prognostic significance in human cancer remain controversial.

Endometrial cancer is currently the most common gynaecologic malignancy in industrialised countries (Amant *et al*, 2005). Although this neoplasm is generally considered non-aggressive, it is a heterogeneous disease with 5-year survival rates ranging from over 80–90% for women with clinical stage I disease (Creutzberg *et al*, 2004). Currently, various clinicopathologic parameters are used to predict the outcome of the disease and to decide the need for adjuvant treatment: surgical stage, histological type, grade, depth of myometrial invasion, cervical stromal invasion, lymph node metastasis, lymph vascular involvement, and peritoneal cytology (Morrow *et al*, 1991; Grigsby *et al*, 1992); however, the majority of parameters have been criticised for their subjectivity and poor reproducibility (Nordstrom *et al*, 1996). Thus, in addition to the conventional clinicopathological parameters, the identification of biochemical or molecular markers more strictly related to the intrinsic biological behaviour of endometrial cancer, and the individualisation of adjuvant therapy based on more reliable prognostic indicators, may be helpful to further improve the survival of patients, as well as to prevent the unnecessary use of adjuvant therapy.

In the present study, we examined GnT-V expression by immunohistochemistry in surgically resected endometrial cancer and analysed its biological and clinical importance, especially as a potential prognostic factor.

## MATERIALS AND METHODS

### Patients and tissue specimens

Seventy-four patients with endometrial endometrioid adenocarcinoma between 1990 and 2005 were included in this study. Initial diagnoses were made preoperatively by the pathological review of endometrial biopsy or curettage specimens. Surgical treatment consisted of total abdominal hysterectomy and bilateral salpingooophorectomy, followed by surgical staging, including peritoneal washing cytology and lymphadenectomy. Patients with histological cell types other than endometrioid adenocarcinoma, such as papillary serous or clear cell, were not included in this study. The mean age of the patients was 58.3 years (range: 38–86). All patients were staged according to the 1988 International Federation of Gynecology and Obstetrics (FIGO) criteria: 43 were stage I (four were IA, 29 were IB, 10 were IC), nine were stage II, 17 were stage III, and five were stage IV. Histological grade was assigned according to the criteria of the World Health Organisation (WHO) classification: 28 were G1 (well differentiated), 33 were G2 (moderately differentiated), and 13 were G3 (poorly differentiated). In this study, all patients with FIGO stage IC and more advanced-stage disease received post-operative adjuvant chemotherapy with six cycles of cisplatin/doxorubicin/cyclophosphamide or cisplatin plus etoposide in 1992–1999, and carboplatin plus paclitaxel after 2000. Patients receiving post-operative radiation therapy or any preoperative treatment were excluded from this study because the number of patients was very small. Tumour recurrence/progression was defined based on clinical, radiological, or histological diagnosis. Patients with recurrence were treated with chemotherapy, local radiation therapy, or surgical tumour resection if possible.

### Western blot analysis

JAR human choriocarcinoma cells were obtained from American Type Culture Collection (ATCC, Manassas, VA, USA) and UtSMC normal human uterine smooth muscle cell line (CC-2562) from Cambrex (Walkersville, MD, USA). Tumour tissue samples and cells were homogenised in a lysis buffer consisting of a protease inhibitor mixture in radioimmunoprecipitation assay buffer. After centrifugation at 15 000 **g** for 20 min, the supernatant was obtained. Twenty micrograms of protein extract was separated by SDS 10% polyacrylamide gel electrophoresis, transferred onto a nitrocellulose membrane, and immunoblotted with anti-GnT-V monoclonal antibody (24D11) (Nakahara *et al*, 2003) at a dilution of 1 : 1000. Immunoreactive proteins were stained using a chemiluminescence detection system (ECL, GE Healthcare, Buckinghamshire, UK).

### Leukoagglutinating phytohaemagglutinin blot analysis

Protein-blotted nitrocellulose filters were prepared in exactly the same way as described for Western blotting. After blocking with 5% skim milk for 30 min at room temperature, the filter was incubated in PBS containing 1 : 1000 diluted biotinylated leukoagglutinating phytohaemagglutinin (L_4_-PHA, Seikagaku, Tokyo, Japan), which preferentially recognises *β*1–6 branches of tri- or tetra-antennary sugar chains, for 1 h at room temperature. The filter was washed three times with PBS containing 0.05% Tween 20 (TPBS) for 10 min each. Substrate binding was detected with a 1 : 1000 dilution of avidin–peroxidase conjugate (ABC kit, Vector Res., CA, USA) in TPBS for 30 min at room temperature. The membrane was washed and then developed using ECL reagents (GE Healthcare, Buckinghamshire, UK).

### Lectin blot analysis on immunoprecipitated *β*1 integrin

For immunoprecipitation, 800 *μ*g of proteins were extracted from each sample tissue. After incubation with 1 *μ*l of anti-human *β*1 integrin mAb MAB2247 (Chemicon International Inc., Temecula, CA, USA) overnight at 4°C, immune complexes were collected with 30 *μ*l of protein G-Sepharose 4EF beads (GE Healthcare, Buckinghamshire, UK). The complexes were released by boiling in sampling buffer without a detergent, separated by 7.5% SDS–PAGE. The membrane filter was analysed by L_4_-PHA lectin blot, as described above. After deprobing and blocking, it was subjected to Western blot analysis using anti-*β*1 integrin mAb as described above.

### Immunohistochemistry for GnT-V

Informed consent was obtained from individual patients for the use of their tissue samples. A mouse monoclonal antibody against recombinant human GnT-V was made according to the standard protocol, as described previously (Murata *et al*, 2000). Briefly, mice were immunised by recombinant GnT-V, and monoclonal antibodies for GnT-V were screened by the availability for immunohistochemistry. The antibody used in the present study recognised ^545^SKNTDFFIGKPTILRELTS^562^ of the human GnT-V amino-acid sequence and gave the best signal for immunohistochemistry. Surgical specimens were fixed in 10% formalin and embedded in paraffin. Paraffin specimens were cut at a thickness of 4 *μ*m. For heat-induced epitope retrieval, deparaffinised sections were soaked in Target Retrieval Solution consisting of 10 mM Tris and 1 mM EDTA (DAKO, Glostrup, Denmark), and treated at 95°C for 30 min in a microwave oven. Immunohistochemical staining was performed using the avidin–biotin immunoperoxidase technique (Histofine SAB-PO kit, Nichirei, Tokyo, Japan). Endogenous peroxidase activity was blocked by incubation with 0.3% H_2_O_2_ in methanol for 15 min, and nonspecific immunoglobulin binding was blocked by incubation with 10% normal goat serum for 10 min. Sections were incubated at 4°C overnight with anti-GnT-V antibody at 1 : 400 dilution or antiproliferating cell nuclear antigen (PCNA) antibody (DAKO) at 1 : 30. The sections were rinsed and incubated for 30 min with the biotinylated second antibody. After washing, the sections were incubated for 5 min with horseradish peroxidase-conjugated streptavidin, and finally treated with 3,3′-diaminobenzidine tetrahydrochloride (Nichirei, Tokyo, Japan) in 0.01% H_2_O_2_ for 3 min. The slides were counterstained with Meyer's haematoxylin. As a negative control, the primary antibody was replaced with normal mouse IgG at an appropriate dilution. As a positive control, tissue sections of normal placenta were used as reported previously (Tomiie *et al*, 2005). *N*-acetylglucosaminyltransferase V expression levels were classified semiquantitatively based on the total scores of the per cent positivity of stained tumour cells and the staining intensity. Namely, the per cent positivity was scored as ‘0’ if <5% (negative), ‘1’ if 5–30% (sporadic), ‘2’ if 30–70% (focal), and ‘3’ if >70% (diffuse) of cells stained, whereas staining intensity was scored relative to the known positive and negative controls as ‘0’ if no staining, ‘1’ if weakly stained, ‘2’ if moderately stained (intermediate level between strong and weak), and ‘3’ if strongly stained. The final GnT-V expression score was defined as follows: ‘GnT-V low’ if the sum of the per cent positivity score and the staining intensity score was 0–4 and ‘GnT-V high’ if the sum was 5–6. In each case, at least three different areas were evaluated. The scoring procedure was carried out twice by two independent observers without any knowledge of the clinical data. The concordance rate was over 95% between the observers. In case of disagreement, the slides were reviewed simultaneously by these two observers, together with another observer, who were seated together at a multiheaded microscope, to resolve the difference of opinion.

### Leukoagglutinating phytohaemagglutinin histochemistry

The expression of *β*1–6 branching asparagine-linked oligosaccharides was analysed by L_4_-PHA histochemistry, with a modified previous method (Suzuki *et al*, 1999). Briefly, after deparaffinisation, trypsinisation was performed in Tris buffer containing 0.1% trypsin (Difco Laboratories, Detroit, MI, USA) and 0.1% CaCl_2_ for 10 min at 37°C after blocking endogenous peroxidase activity. The sections were incubated with 5% skim milk in PBS for 20 min at room temperature to block nonspecific staining. The sections were incubated with HRP-PHA-L_4_ (Seikagaku) at a dilution of 1 : 200 at 4°C overnight. Staining was performed by the biotin–streptavidin peroxidase method with 3,3′-diaminobenzidine as a chromogen. Haematoxylin was used as a counterstain.

### Statistical analysis

Statistical analysis was performed using *χ*^2^ for the independence test, Fisher's exact probability test, or Student's *t*-test. For survival analysis, the Kaplan–Meier method was applied, and statistical significance was calculated using the log-rank test. Cox proportional-hazard analysis was used for univariate and multivariate analyses to explore the effect of variables on survival. StatView software ver.5.0 (SAS Institute Inc., Cary, NC, USA) was used for statistical analyses, and a *P*-value of <0.05 was considered significant.

## RESULTS

### GnT-V protein expression and lectin blot analysis in endometrial cancer tissue

**Figure 1:**
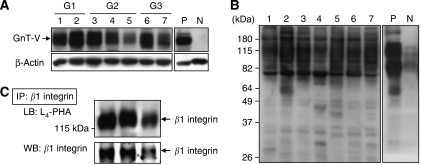
GnT-V and *β*1–6 branching glycoprotein expression, and *β*1–6GlcNAc branching of *β*1 integrin in endometrial cancer tissues. (**A**) Western blot analysis with anti-GnT-V mAb. (**B**) Lectin blot analysis with L_4_-PHA. Lanes 1–7 corresponded to seven different endometrial cancer patients (G1, grade 1; G2, grade 2; G3, grade 3). P, JAR is a choriocarcinoma cell line used as a positive control for GnT-V expression; N, normal human uterine smooth muscle cells (UtSMC) as a negative control. (**C**) *β*1 integrin was immunoprecipitated from endometrial cancer tissues from three different patients. The amount of *β*1–6GlcNAc branching of *β*1 integrin was analysed by means of an L_4_-PHA lectin blot (upper panel). The membrane was reprobed with a specific mAb to *β*1 integrin (lower panel).

First, the GnT-V protein expression was examined in endometrial cancer tissues obtained from seven patients using Western blot analysis. In all samples, GnT-V protein was detected as approximately 110 kDa bands, although its expression level varied among the samples (Figure 1A). The broad bands of GnT-V may be due to oligosaccharide modification of GnT-V, because GnT-V has as many as five sugar chains and the molecular weight of GnT-V is smaller with less *β*1–6GlcNAc branching (Nakahara *et al*, 2003).

To evaluate the level of *β*1–6 branching, we also performed lectin blot analysis on total cellular proteins using L_4_-PHA, which preferentially binds to GlcNAc residues on *β*1–6 branches of tri- or tetra-antennary sugar chains (Figure 1B). This analysis showed that GnT-V certainly catalysed such specific glycosylation to target glycoproteins, whose major molecular sizes were approximately 60–200 kDa.

### Lectin blot analysis on immunoprecipitated *β*1 integrin

It has been reported that *β*1 integrin is a target molecule of GnT-V in certain cell lines (Guo *et al*, 2002; Nakahara *et al*, 2003); therefore, to investigate the glycosylation state of *β*1 integrin, we performed L_4_-PHA blot analysis on immunoprecipitated *β*1 integrin. The results showed that *β*1 integrin, which had been immunoprecipitated from endometrial cancer tissues, certainly contained *β*1–6 GlcNAc branching (Figure 1C), suggesting that *β*1 integrin is a target substrate of GnT-V in endometrial cancers.

### Immunohistochemical expression of GnT-V and L_4_-PHA staining in endometrial cancer tissues

**Figure 2:**
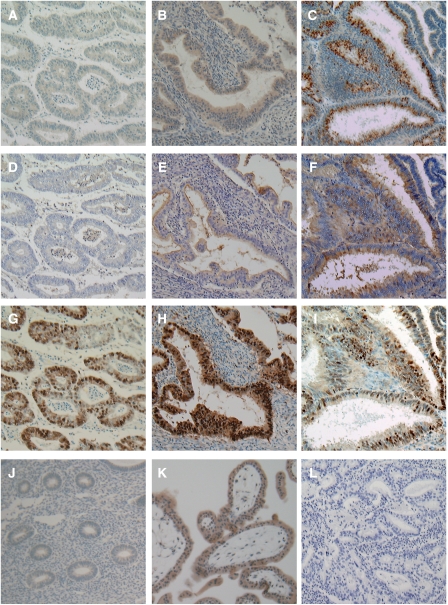
Immunohistochemical staining patterns for GnT-V and staining of L_4_-PHA in endometrial cancers. Staining pattern of a tumour: (**A**) GnT-V low; (**B** and **C**) GnT-V high. (**D**–**F**) L_4_-PHA staining and (**G**–**I**) PCNA immunostaining were performed simultaneously with the same **A**, **B**, and **C** specimens, respectively. (**J**) Normal endometrial cells showed very faint or negative GnT-V expression. (**K**) Positive control for GnT-V (normal placenta). (**L**) Negative control. Original magnification, × 100.

We examined GnT-V expression in endometrial cancer tissues by immunohistochemical staining using 74 surgical specimens. As shown in Figure 2A–C, GnT-V immunoreactivity was detected at variable levels, and was found in the cytoplasm of cancer cells, which were identified using PCNA co-staining (Figure 2G–I). In contrast, GnT-V immunoreactivity was very faint or absent in tumour stroma (Figure 2A–C) and normal endometrium (Figure 2J). *N*-acetylglucosaminyltransferase V immunoreactivity was not detected in the negative control experiment (Figure 2L), whereas it was strongly detected in placental tissues used as a positive control (Figure 2K). Next, we examined the expression of *β*1–6 branching asparagine-linked oligosaccharides by L_4_-PHA histochemistry in the same sections, simultaneously. Leukoagglutinating phytohaemagglutinin staining was very weak in tumour cells that showed weak GnT-V immunostaining (Figure 2D), while it was moderate-to-strong in cancer cells that showed high GnT-V immunostaining (Figure 2E and F). These results were consistent with the results of lectin blotting, which recognised *β*1–6 branching in endometrial cancer with variable intensity.

### Correlation of GnT-V expression with clinicopathological parameters

Of the 74 specimens examined, ‘low GnT-V expression’ tumours were found in 31 (41.9%) cases, and ‘high GnT-V expression’ in 43 (58.1%) cases, respectively. The correlations of high GnT-V expression with various clinicopathological parameters in the 74 cases are summarised in Table 1. High GnT-V expression was positively correlated with age (*P*=0.045), histological grade (*P*=0.011), and lymph vascular space involvement (*P*<0.001), but not with the FIGO surgical stage, lymph node metastasis, and myometrial invasion.

### Correlation of GnT-V expression with patient survival

**Figure 3:**
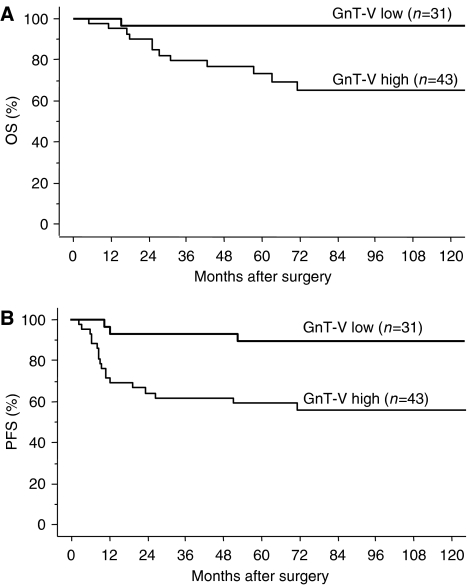
Overall survival (OS) and progression-free survival (PFS) curve drawn using the Kaplan–Meier method according to GnT-V expression in endometrial cancer patients. OS (**A**) and PFS (**B**) in all patients (*n*=74). Significant differences in OS (*P*=0.0041), and PFS (*P*=0.0023).

Follow-up data were available for all 74 patients and the median follow-up period was 72.9 months (range: 3–160). During the follow-up period, disease progression/recurrence was observed in 20 patients (27.0%), of which 12 (16.2%) died. The median time to progression/recurrence and death was 15.0 and 28.5 months, respectively. To evaluate the impact of GnT-V expression on patient prognosis, overall survival (OS) and progression-free survival (PFS) curves were constructed using the Kaplan–Meier method. The OS rates of patients with GnT-V low and GnT-V high were 96.8 and 74.4%, respectively (Figure 3A). The 5-year PFS rates for GnT-V low and GnT-V high were 90.3 and 60.5%, respectively (Figure 3B). Patients with high GnT-V expression had significantly impaired OS (*P*=0.0041) and PFS (*P*=0.0023) as compared to patients with low expression of GnT-V (Figure 3A and B).

### Multivariate analysis of prognostic variables in endometrial cancer patients

Cox proportional-hazard analysis was performed to compare the impact of GnT-V expression on survival with currently used clinicopathological prognostic factors. The results of univariate/multivariate analyses of the variables, including GnT-V expression, age, surgical stage, grade, lymph vascular space involvement, node status, and myometrial invasion, with respect to OS and PFS, are shown in Tables 2 and 3, respectively. Among the seven variables, there was no significant prognostic factor with respect to OS on multivariate analysis, although the surgical stage, lymph vascular invasion, node status, myometrial invasion, and GnT-V expression were significant prognostic factors on univariate analysis (Table 2). In contrast, only GnT-V expression was found to be an independent prognostic factor (hazard ratio=4.164, *P*=0.0364) with respect to PFS on multivariate analysis (Table 3).

## DISCUSSION

In the present study, we demonstrated the expression of GnT-V in endometrial cancer using 74 surgical specimens, and found that high GnT-V expression by tumour cells was positively correlated with impaired clinical outcome. The immunoreactivity of GnT-V was very weak in normal endometrium and increased clearly in endometrial cancer. Oligosaccharides on glycoproteins are altered in tumorigenesis and often play a role in the regulation of the biological characteristics of tumours (Hakomori, 1989). Each oligosaccharide is synthesised by a specific glycosyltransferase whose expression affects a specific function of glycoprotein through glycosylation in normal and malignant cells (Varki, 1993). Among many glycosyltransferases and oligosaccharides, GnT-V and its products, *β*1–6 branching *N*-linked oligosaccharides, have been associated with the malignant potential of cancer (Dennis *et al*, 1987). In colon cancer, breast cancer, and oesophageal cancer, GnT-V expression has a positive correlation with poor prognosis, which is consistent with our results in the present study (Fernandes *et al*, 1991; Murata *et al*, 2000; Ishibashi *et al*, 2005). On the other hand, low GnT-V expression is associated with shorter survival and poor prognosis in non-small cell lung cancer, bladder cancer, and hepatocellular cancer (Ito *et al*, 2001; Dosaka-Akita *et al*, 2004; Ishimura *et al*, 2006). It may depend on the type of cancer or originating tissues whether GnT-V expression is associated positively with poor prognosis.

We confirmed the levels of *β*1–6 branching in endometrial cancers using lectin blotting and L_4_-PHA histochemistry. *N*-acetylglucosaminyltransferase V expression is not equal to the expression of *β*1–6 branching asparagine-linked oligosaccharides analysed by L_4_-PHA histochemistry (Dosaka-Akita *et al*, 2004). This is because GnT-V has been shown to function as an inducer of angiogenesis (Saito *et al*, 2002), which is completely different from the original function of glycosyltransferase, and GnT-V expression does not necessarily result in the synthesis of *β*1–6 branching oligosaccharides. Our results showed that GnT-V-expression intensity was well consistent with L_4_-PHA-staining intensity in tumour cells. These findings suggested that GnT-V plays a functional role in the malignant potential of endometrial cancer cells by the synthesis of *β*1–6 branching oligosaccharides.

Our lectin blotting revealed that major target glycoproteins of GnT-V in endometrial cancer were 60–200 kDa in molecular size. Previous reports indicated several specific substrates for GnT-V glycosylation and changes in the biological characteristics of cancer cells. An increased level of *β*1–6 branching on *β*1 integrin, a 130 kDa subunit of fibronectin receptor, by GnT-V resulted in the inhibition of cisplatin-induced apoptosis, or inhibition of clustering of *α*5*β*1 integrin and promotion of cell migration in neck squamous cell carcinoma and fibrosarcoma (Guo *et al*, 2002; Nakahara *et al*, 2003). Lamp-1 is a 90–120 kDa molecule expressed on cell and lysosome membranes, and plays an important role in lysosomal trafficking, matrix degradation, and cell adhesion. *N*-acetylglucosaminyltransferase V glycosylation of lamp-1 inhibits its degradation, and the stabilisation of lamp-1 results in increased extracellular matrix degradation (Fukuda, 1991; Kundra and Kornfeld, 1999). Matriptase is an 80 kDa serine protease involved in cancer metastasis by the activation of urokinase-type plasminogen activator (u-PA) and hepatocyte growth factor (Lee *et al*, 2000). The addition of *β*1–6 branching on matriptase by GnT-V inhibits its degradation, resulting in the upregulation of matriptase expression in gastric cancer (Ihara *et al*, 2002). Of those molecules, matriptase and *β*1 integrin were expressed in endometrial cancer, especially *β*1 integrin with *β*1–6 branching by GnT-V (Figure 1C). Increased GnT-V did not change the expression of *α*5*β*1 integrin, but increased the level of *β*1–6 branching on it, and subsequently inhibited integrin clustering and signal transduction pathways. As a result, cell migration and invasion were stimulated (Guo *et al*, 2002; Nakahara *et al*, 2006). In the present study, we showed that high GnT-V expression was significantly correlated with lymph vascular invasion and the histological grade. These results suggested that GnT-V might be involved in tumour cell migration or invasion by the modification of oligosaccharides of *β*1 integrin in endometrial cancer, resulting in disease progression and poor prognosis. In addition, GnT-V might be linked to malignant potential, increasing *β*1–6 branching synthesis in poorly differentiated cancer cells; however, the functional significance of GnT-V expression in endometrial cancer has to be studied further.

In conclusion, we demonstrated that high GnT-V expression correlated with impaired clinical outcome in endometrial cancer patients. Furthermore, GnT-V was an independent prognostic factor for PFS. These results indicate that GnT-V is a reliable and promising prognostic indicator and might become a novel molecular target in the strategy for the treatment of endometrial cancer.

## Figures and Tables

**Table 1:** Correlation of GnT-V expression with clinicopathological factors in endometrial cancer

		**GnT-V expression**		
**Characteristics**	**Patient no.**	**Low**	**High**	***P*-value^a^**
All cases	74	31 (41.9%)	43 (58.1%)	
*Age*				
⩽60	45	23 (51.1%)	22 (48.9%)	0.045
>60	29	8 (27.6%)	21 (72.4%)	
				
*FIGO surgical stage*				
I+II	52	25 (48.1%)	27 (51.9%)	0.097
III+IV	22	6 (27.3%)	16 (72.3%)	
				
*Histological grading*				
G1	28	17 (60.7%)	11 (39.3%)	0.011
G2/G3	46	14 (30.4%)	32 (69.6%)	
				
*Lymph vascular invasion*				
Negative	40	24 (60.0%)	16 (40.0%)	<0.001
Positive	34	7 (20.6%)	27 (79.4%)	
				
*Nodal status*				
N0	60	27 (45.0%)	33 (55.0%)	0.262
N1	14	4 (28.6%)	10 (71.4%)	
				
*Myometrial invasion*				
1/2>	43	20 (46.5%)	23 (53.5%)	0.343
1/2⩽	31	11 (35.5%)	20 (64.5%)	

Abbreviations: FIGO=International Federation of Gynecology and Obstetrics; GnT-V=*N*-acetylglucosaminyltransferase V.

a *χ*^2^-test.

**Table 2:** Univariate and multivariate analyses of overall survival in endometrial cancer patients

			**Multivariate analysis**		
**Variables**	**Categories**	**Univariate *P* value^a^**	**HR**	**95% CI**	***P*-value**
Age	⩽60				
	>60	0.8046	—	—	—
FIGO surgical stage	I+II				
	III+IV	<0.0001	2.922	0.639–13.362	0.1667
Histological grading	G1				
	G2/G3	0.1425	—	—	—
Lymph vascular invasion	Negative				
	Positive	0.0004	1.872	0.261–13.446	0.5329
Nodal status	N0				
	N1	<0.0001	3.466	0.940–12.787	0.062
Myometrial invasion	1/2>				
	1/2⩽	0.011	1.236	0.292–5.224	0.7734
GnT-V	Low				
	High	0.0041	6.053	0.649–56.452	0.5329

Abbreviations: CI=confidence interval; FIGO=International Federation of Gynecology and Obstetrics; GnT-V=*N*-acetylglucosaminyltransferase V; HR=hazard ratio.

a Log-rank test.

**Table 3:** Univariate and multivariate analyses of progression-free survival in endometrial cancer patients

			**Multivariate analysis^a^**		
**Variables**	**Categories**	**Univariate *P* value^a^**	**HR**	**95% CI**	***P*-value**
Age	⩽60				
	>60	0.8643	—	—	—
FIGO surgical stage	I+II				
	III+IV	<0.0001	3.049	0.997–9.327	0.0507
Histological grading	G1				
	G2/G3	0.0625	—	—	—
Lymph vascular invasion	Negative				
	Positive	0.0003	1.352	0.350–5.222	0.6616
Nodal status	N0				
	N1	<0.0001	2.242	0.789–6.365	0.1295
Myometrial invasion	1/2>				
	1/2⩽	0.0048	1.386	0.441–4.356	0.5761
GnT-V	Low				
	High	0.0023	4.164	1.095–15.840	0.0364

Abbreviations: CI=confidence interval; FIGO=International Federation of Gynecology and Obstetrics; GnT-V=*N*-acetylglucosaminyltransferase V; HR=hazard ratio.

a Log-rank test.
